# The mitotic cell cycle‐associated nomogram predicts overall survival in lung adenocarcinoma

**DOI:** 10.1002/cam4.6676

**Published:** 2023-11-06

**Authors:** Xu He, Huafu Zhou, Qianyu Huang, Yue Li

**Affiliations:** ^1^ Department of Cardio‐Thoracic Surgery The First Affiliated Hospital of Guangxi Medical University Nanning China; ^2^ Department of Respiratory and Critical Care Medicine The First Affiliated Hospital of Guangxi Medical University Nanning China

**Keywords:** lung adenocarcinoma, mitotic cell cycle, mutant score, prognosis prediction, TP53 mutant

## Abstract

**Background:**

This study aimed to develop a prognostic model for lung adenocarcinoma (LUAD) associated with mitotic cell cycle. The model will predict the probability of survival at different time points and serve as a reference tool to evaluate the effectiveness of LUAD treatment.

**Methods:**

A cohort of 442 patients with LUAD from the gene expression omnibus (GEO) database was randomly divided into a training group (*n* = 299) and a validation group (*n* = 99). The least absolute shrinkage and selection operator (LASSO)‐COX algorithm was used to reduce the number of predictors based on the clinicopathological and RNA sequencing data to establish mutant characteristics that could predict patient survival. Additionally, gene ontology (GO), Kyoto Encyclopedia of Genes and Genomes (KEGG), gene set variation analysis (GSVA), and gene set enrichment analysis (GSEA) analyses were conducted on the mutant characteristics. The performance of the developed nomogram was evaluated using calibration curves and the *C*‐index.

**Results:**

The mutant characteristics had prognostic value for LUAD and acted as an independent prognostic factor. The mutant characteristics profile derived from the LASSO‐COX algorithm demonstrated a significant association with overall survival in patients with LUAD. Functional annotation based on the mutant score, its involvement in the phase transition of the mitotic cell cycle, and its regulatory processes. The nomogram, which combined the mutant score with clinical factors associated with prognosis, showed robust accuracy in both the training and validation groups.

**Conclusion:**

This study presents the first individualized model that establishes a mutant score for predicting survival in LUAD. This model can be used as a predictive tool for determining 1‐, 2‐, 3‐, and 5‐year survival probabilities in patients with LUAD.

## Abstract 2

This study presents the first individualized model that establishes a mutant score for predicting survival in LUAD. This model can be used as a predictive tool for determining 1‐, 2‐, 3‐, and 5‐year survival probabilities in patients with LUAD.

## 1 INTRODUCTION

Although there are various preventive treatments for lung cancer, such as early lung cancer detection, immune checkpoint inhibitors, targeted therapies, and surgical interventions.^1^, ^2^, ^3^ In 2023 alone, an estimated 127,070 patients may succumb to lung cancer, with 238,340 new cases resulting in death. This number is 2.5 times higher than the second leading cause of cancer‐related deaths, like colorectal cancer. Among the histologic subtypes of lung cancer, lung adenocarcinoma (LUAD) is the most prevalent, accounting for a concerning 38.5% of all cases.^4^, ^5^, ^6^, ^7^

Data from the Surveillance, Epidemiology, and End Results (SEER) database indicate that the median survival of LUAD ranges from 1 to 6 years across different stages (T1 to T4). For the N0 stage, the median survival is less than 5 years, whereas for the N3 stage, it is even less than 10 months. Despite the slower growth of LUAD compared to squamous lung cancer, it still poses a significant threat to patient survival.^8^, ^9^ Therefore, the development of a highly accurate prognostic model for LUAD, specifically regarding overall survival (OS), would greatly assist clinicians in monitoring patients with LUAD and devising appropriate treatment plans to improve prognosis.

Nomograms have become widely used in predicting OS and progression‐free survival (PFS) in various cancers, including esophageal, renal, nasopharyngeal, and neuroendocrine tumors.^10^, ^11^ Moreover, the gene expression omnibus (GEO) database provides a vast collection of clinical, sequencing, and corresponding follow‐up data for LUAD. This extensive dataset offers a promising possibility for constructing an OS prediction model for LUAD.

In addition to relying on the well‐known “Warburg effect” for energy production to support tumor cell growth,^12^ tumor cells also use lactic acid to promote their own proliferation and evade immune responses. Elevated levels of lactic acid are strongly associated with tumor metastasis, recurrence, and drug resistance.^13^, ^14^ Similarly, tumor cells use glutamine to suppress oxidative stress, maintain mitochondrial membrane integrity, and support the survival of proliferating cells. Glutamine metabolism also plays a role in enhancing anti‐tumor inflammatory immune responses and participating in the activation of effector T cells.^15^, ^16^, ^17^ These findings underscore the critical role of metabolic processes in tumor development. Furthermore, it has been demonstrated that phospholipid dephosphorylation, a metabolic pathway, is involved in driving carcinogenesis.^18^ However, there is a lack of studies investigating the relevance of this metabolic pathway in LUAD. Therefore, we aimed to investigate the association between phospholipid dephosphorylation and LUAD and use it to construct an OS model that incorporates these functions and can be applied to LUAD.

The aim of this study was to investigate the biological characteristics associated with phospholipid dephosphorylation in LUAD and develop a prognostic model to predict survival probabilities at different time points. This model will serve as a valuable reference tool for evaluating the efficacy of treatment strategies in LUAD.

## 2 METHODS

### 2.1 Dataset collection and process

The RNA sequencing data for 442 patient samples with LUAD were retrieved from the GSE72094 dataset in the GEO database.^19^ The data were randomized using the car and survivor packages in the R language. A total of 299 patients with LUAD were assigned to the training group, while the remaining 99 patients were assigned to the validation group.

The sequencing, clinical, and follow‐up data of patients with LUAD were previously uploaded to the GEO database by the corresponding authors. All the data used or analyzed in this study are accessible from the corresponding authors upon request, and no additional ethics committee approval was required for their usage.

The gene lists for each biological function were recently obtained from the gene set enrichment analysis (GSEA) network (http://www.gsea‐msigdb.org/gsea/downloads.jsp), and the AmiGo2 website (http://amigo.geneontology.org/amigo) was utilized to download relevant data.

### 2.2 Gene ontology (GO) and gene set variation analysis (GSVA) enrichment scores for biological processes

Biological functional enrichment scores were calculated for each patient using GSVA based on RNA transcriptome sequencing data with LUAD. The default parameters of the GSVA package in R was used in GSVA analysis. Heatmaps depicting the enrichment results were generated using the heatmap package. A Pearson correlation analysis was conducted to examine the correlation between mutant scores and the different phases of the mitotic cell cycle. Furthermore, a list of the most relevant genes associated with the mutant score was uploaded to the DAVID database. Official gene symbols were chosen as identifiers, and *Homo sapiens* was selected as the species. Subsequently, GO analysis and Kyoto Encyclopedia of Genes and Genomes (KEGG) pathway analysis were performed, and the top five results with the lowest *p*‐value (*p* < 0.05) are presented in this study.

### 2.3 LASSO‐COX dimension reduction analysis of dataset

The least absolute shrinkage and selection operator (LASSO)‐COX dimension reduction analysis was conducted using R package which named glmnet and survival, and to facilitate accurate parameter estimation, the coefficients of unimportant genes within the dataset were set to zero through compression. Simultaneously, dimension reduction was performed to analyze the down‐scaled genes. Subsequently, these genes were screened to develop a COX clinical prediction model and form the basis for the construction of the Nomogram. The best lambda (*λ*) value in this study was determined by selecting the *λ* value matching to the minimum bias. Ultimately, a total of 13 candidate genes and their corresponding *λ* values were identified based on the OS of patients with LUAD in the training group. The candidate genes and their *λ* values are as follows: *CHRM5*: −0.756681086, *SACM1L*: −0.590029725, *INPP5J*: −0.565327555, *INPPL1*: −0.378109854425639, *INPP5B*: −0.290441078449763, *INPP5E*: −0.263468329758548, *INPP5K*: −0.216071265904673, *SYNJ1*: −0.189221770880062, *EPHX2*: −0.166152094252374, *INPP5D*: −0.148523596699476, *SGPP2*: −0.0565191508271845, *INPP4B*: 0.220746430109358, *INPP5A*: 0.296246301201646).

To calculate the mutant score for each LUAD patient, the following formula was used:

$$\begin{array}{cc} \text{Mutantscore}= & \text{exprCHRM}5\times \lambda_{\text{CHRM}5}+\text{exprEPHX}2\times \lambda_{\text{EPHX}2}\\ & +\text{exprINPP}4B\times \lambda_{\text{INPP}4B}+\text{exprINPP}5A\times \lambda_{\text{INPP}5A}\\ & +\text{exprINPP}5B\times \lambda_{\text{INPP}5B}+\text{exprINPP}5D\times \lambda_{\text{INPP}5D}\\ & +\text{exprINPP}5E\times \lambda_{\text{INPP}5E}+\text{exprINPP}5J\times \lambda_{\text{INPP}5J}\\ & +\text{exprINPP}5K\times \lambda_{\text{INPP}5K}+\text{exprINPPL}1\times \lambda_{\text{INPPL}1}\\ & +\text{exprSACM}1L\times \lambda_{\text{SACM}1L}+\text{exprSGPP}2\times \lambda_{\text{SGPP}2}\\ & +\text{exprSYNJ}1\times \lambda_{\text{SYNJ}1}. \end{array}$$

In the formula, “expr_gene_” represents the expression standard of the corresponding gene, and λ_gene_” represents the matching *λ* value for that gene.

### 2.4 Nomogram construction

In the training group, a nomogram was constructed for analysis using the rms package in R. The nomogram consisted of two parts: the scoring system in the above part and the prediction system in the lower part. The total score and individual factor scores from the nomogram could accurately predict the 1‐, 2‐, 3‐, and 5‐year survival of patients with LUAD. To validate the prediction accuracy of OS, the nomogram was applied to the validation group of patients. The calibration curves and *C*‐index values were used to assess the accuracy of survival prediction.

### 2.5 Statistical analysis

Statistical analyses were conducted using the following software: R (version 4.21, available at https://www.r‐project.org/), IBM SPSS Statistics for Windows (version 26, IBM Corp.), and GraphPad Prism (version 8.0). Prognostic values were calculated using COX and Kaplan–Meier analyses. GSEA analysis was performed using the limma, org.Hs.eg.db, cluster profiler, and enrich plot packages in R. GO was performed using DAVID (available at https://david.ncifcrf.gov/). A significance level of *p* < 0.05 was considered statistically significant for all analyses.

## 3 RESULTS

### 3.1 Prognostic value of phospholipid dephosphorylation in LUAD

**FIGURE 1:**
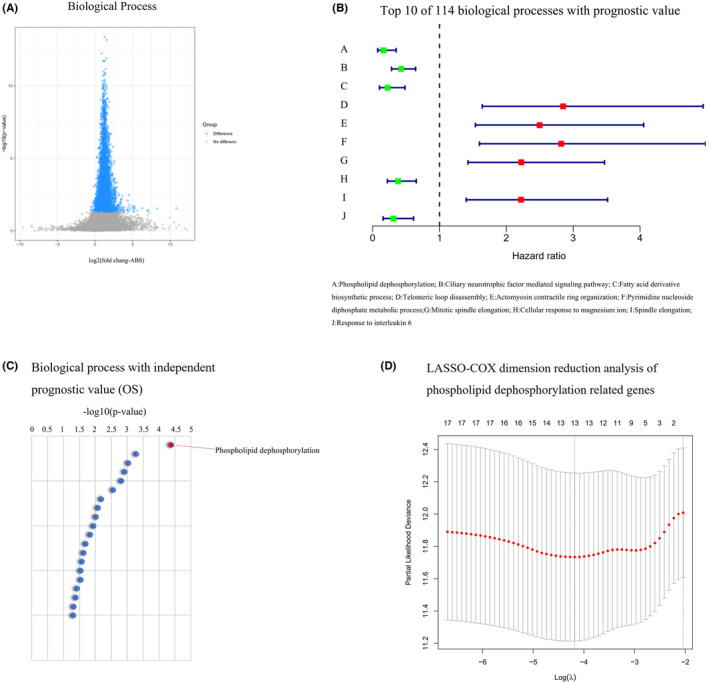
Biological processes that differ between *TP53* wild‐type and mutant in LUAD and establishment of the mutant fraction through LASSO‐COX analysis. (A) Gray indicates no significant change in biological processes, while blue represents significantly elevated biological processes. (B) Top 10 of 114 biological processes with prognostic value ranked from smallest to largest *p*‐value. (C) A phospholipid dephosphorylation showed the highest significance among the 25 BPs with independent prognostic value in LUAD. (D) LASSO‐COX analysis identified the 13 most representative genes related to phospholipid dephosphorylation. ABS, absolute value; LASSO, least absolute shrinkage and selection operator; LUAD, lung adenocarcinoma.

Patients with LUAD were categorized into two groups based on the presence or absence of *TP53* mutations. Using the GSVA algorithm, we calculated 7745 biofunctional enrichment scores for patients with LUAD in the training group, consisting of 226 *TP53* wild‐type and 73 mutant *TP53*. Analyses revealed that 3804 biological processes exhibited significant differences between *TP53* wild‐type and mutant LUAD (Figure 1A). We applied the same calculation method to the validation group, comprising 99 patients, and identified 114 identical biological processes that exhibited prognostic value for OS using batch univariate COX calculations. Further classification of these 114 biological processes showed that cell cycle (7%) and metabolic processes (13%) accounted for a significant proportion of the biological processes associated with proliferation, transcription, translation, metabolic processes, response to stimuli, and immune response in tumor development. Notably, the phospholipid dephosphorylation function was also found among the 114 biological processes with prognostic value for LUAD, as anticipated.

### 3.2 Development of the *TP53* mutant profile in LUAD

The 114 biological processes with prognostic value were subjected to multifactorial COX analysis, resulting in the identification of 25 biological processes that exhibited independent prognostic significance. Notably, the phospholipid dephosphorylation metabolic process was found to be significantly inhibited in patients with LUAD with mutant *TP53*, and it showed the strongest independent prognostic value for OS (Figure 1B,C). Subsequently, a TP53 mutational fraction was constructed based on mutations in genes associated with the phospholipid dephosphorylation metabolic process using LASSO‐COX dimensional reconstruction analysis (Figure 1D). From the analysis, a total of 13 candidate genes (*CHRM5*, *SACM1L*, *INPP5J*, *NPPL1*, *INPP5B*, *INPP5E*, *INPP5K*, *SYNJ1*, *EPHX2*, *INPP5D*, *SGPP2*, *INPP4B*, and *INPP5A*) were selected, and their matching *λ* values were used to calculate the mutant score for each patient. The median mutant score obtained from the training group (−21.7691) was defined as the critical value.

### 3.3 Stable mutant fraction in prediction of the prognosis of LUAD

**FIGURE 2:**
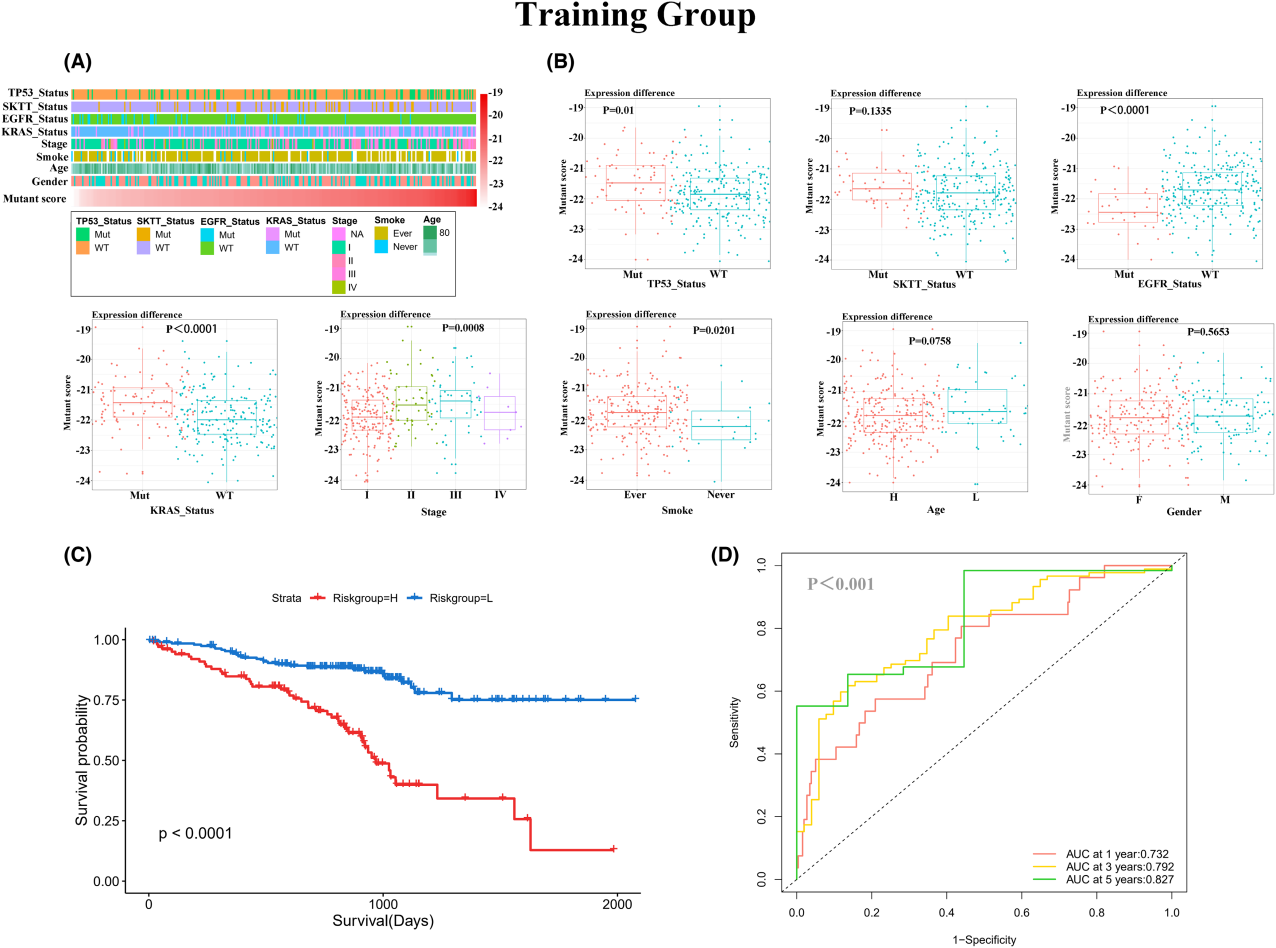
Relationship between mutant fraction and clinicopathological characteristics and survival of patients with LUAD in the training group. (A) A heatmap showing the association between clinicopathological characteristics and mutant fraction in the training group. (B) A box plot showing the distribution of the mutant fraction in the training group. The significance of the difference between two groups was verified using the Mann–Whitney test, and the significance of the difference among the three groups or more groups was verified using the Kruskal–Wallis test. (C) Kaplan–Meier curves showing that patients in the high‐risk group had shorter OS compared to those in the low‐risk group. (D) The ROC curves showing the superior accuracy of the mutant fraction as a predictive marker. LUAD, lung adenocarcinoma; OS, overall survival; ROC, receiver operating curve.

**FIGURE 3:**
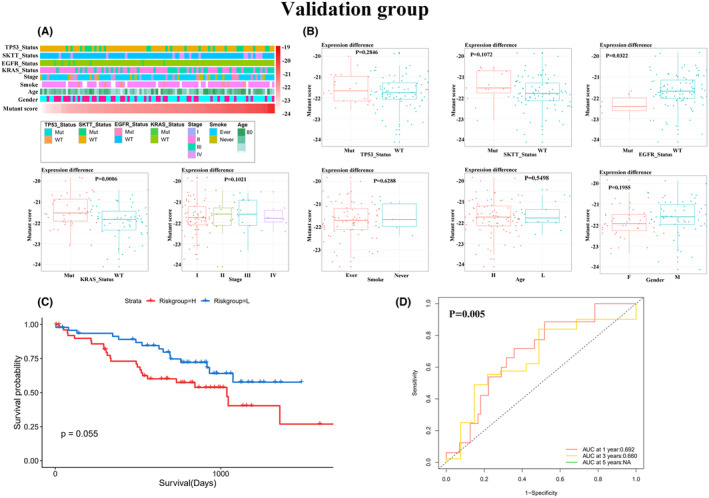
Relationship between mutant fraction and clinicopathological characteristics and survival of patients with LUAD in the validation group. (A) A heatmap showing the relationship between clinicopathological characteristics and mutant fraction in the validation group. (B) A box plot showing the distribution of mutant fraction in the validation group based on different clinicopathological characteristics. The significance of the difference between two groups was verified using the Mann–Whitney test, and the significance of the difference among the three groups was verified using the Kruskal–Wallis test. (C) Kaplan–Meier curves showing that patients in the high‐risk group had shorter OS compared to those in the low‐risk group. (D) The ROC curves showing the superior accuracy of the mutant fraction as a predictive marker.

Patients in different mutation risk groups exhibited varying clinicopathological characteristics. In the training group, the mutation status of *TP53*, *EGFR*, and *KRAS* showed distinct distribution patterns with increasing mutation scores (Figure 2A). Similarly, in the validation group, the mutation status of *TP53* and *KRAS* showed distinct characteristics across different mutation risk groups (Figure 3A). Furthermore, both in the training and validation groups, the mutant fraction demonstrated excellent predictive value for OS (Figures 2C and 3C), with the highest annual survival area under the curve (AUC) reaching 82% in the training group and 69% in the validation group (Figures 2D and 3D).

### 3.4 Association between mutant score of the mutant signature and clinicopathological characteristics

Further investigation of the association between the mutant score and clinicopathological factors revealed interesting findings. In the training group, the mutant score exhibited a significant increase in patients with *TP53* and *KRAS* mutations, as well as in smokers and those in stages II and III (Figure 2B). Conversely, in the validation group, a significant increase in the mutant score was observed only in patients with *KRAS* mutations (Figure 3B). However, in both the training and validation groups, no significant correlation was found between the mutant score and age (60 years as the cut‐off), gender, or the mutation status of SKTT.

### 3.5 Association between mutant score and the mitotic cell cycle

**FIGURE 4:**
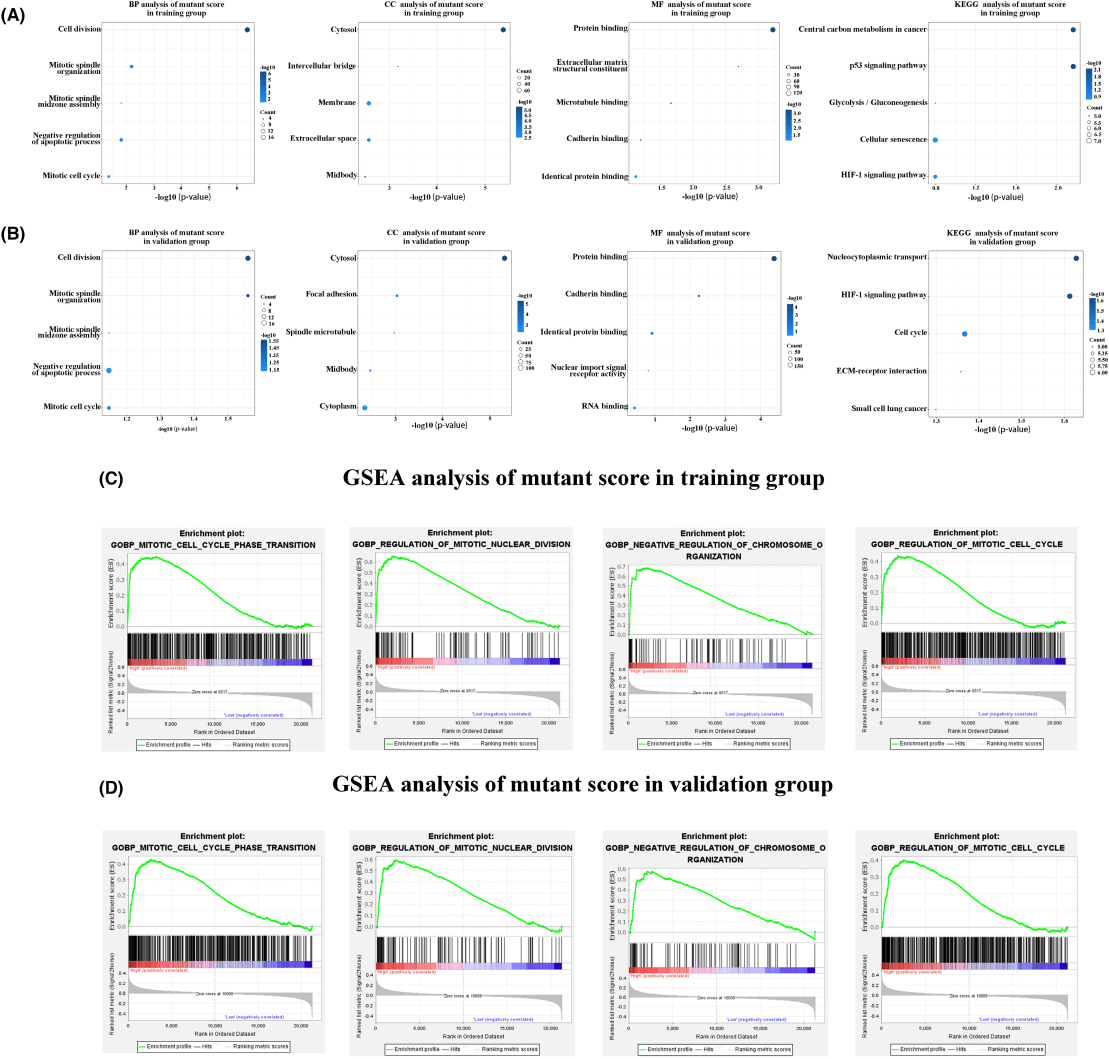
Biological processes associated with the mutant fraction. (A, B) Biological processes and pathways associated with the mutant fraction identified by GO and KEGG analysis in the training and validation groups. (C, D) GSEA analysis revealing the close association between mutant fraction and mitotic cell cycle phase transition and regulation in the training and validation groups. GO, gene ontology; GSEA, gene set enrichment analysis; KEGG, Kyoto Gene and Genome Encyclopedia.

To gain further insights into the biological processes and pathways associated with mutant score, we conducted GO, KEGG, and GSEA analyses. By focusing on the genes that exhibited the strongest associations with mutant scores, we performed GO and KEGG analyses using these specific genes. The GO analysis revealed that mutant scores were significantly associated with biological processes such as cytokinesis, mitotic spindle organization, and the mitotic cell cycle in both the training and validation groups. Furthermore, the KEGG analysis demonstrated a close correlation between mutant scores and the HIF‐1 signaling pathway (Figure 4A,B). Subsequently, GSEA conducted on the training and validation groups provided additional validation of the strong association between mutant scores and functions associated with the phase transition and the regulation of the mitotic cell cycle (Figure 4C,D).

### 3.6 A close association between the mutant scores and the phase transition of the mitotic cell cycle and its regulatory processes

**FIGURE 5:**
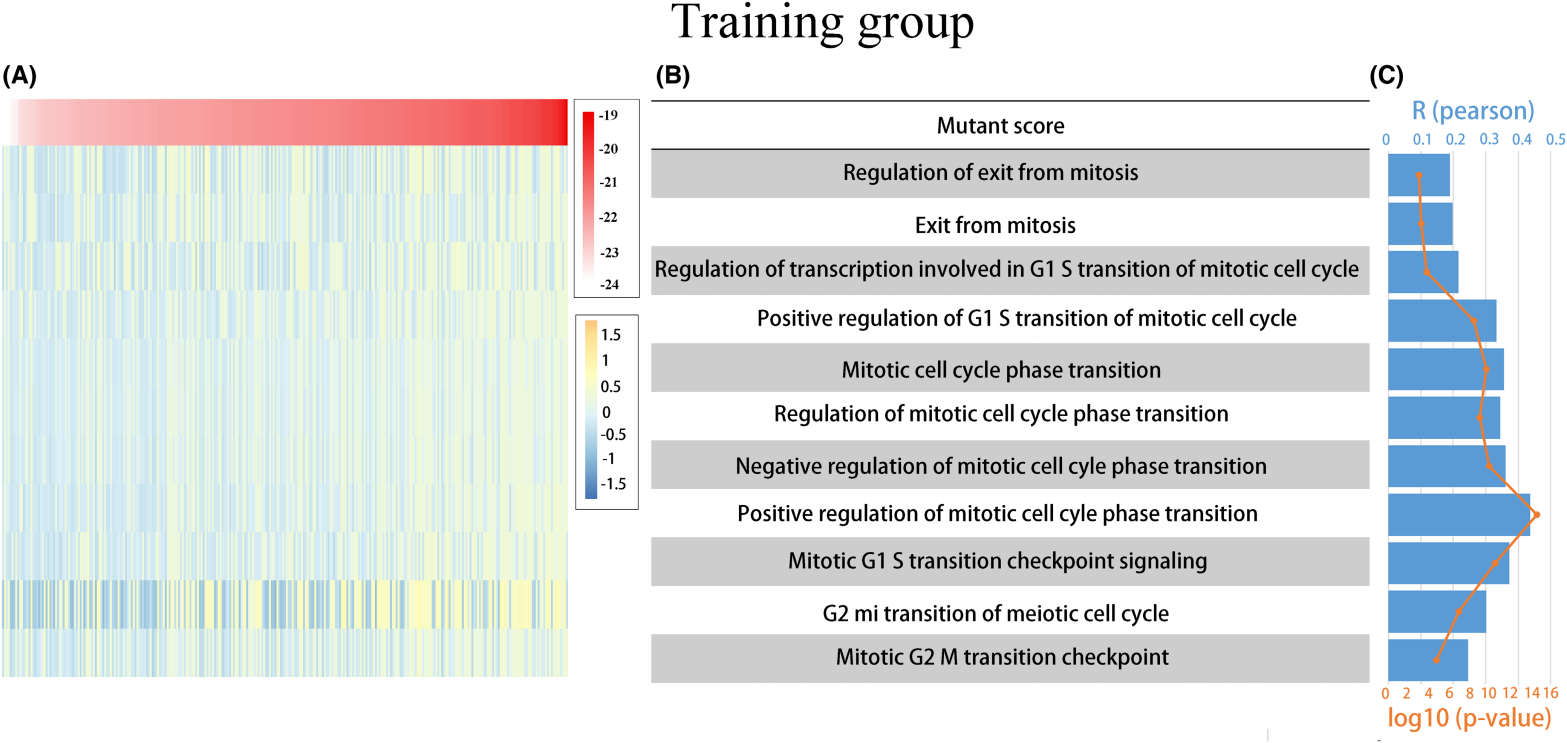
Correlation analysis of mutant scores with mitotic cell cycle enrichment scores. (A) A heatmap showing the expression of mutant fraction and mitotic cell cycle enrichment scores for each patient in the training and validation groups, sorted from highest to lowest mutant fraction. (B, C) Right‐hand bar and line graphs showing the *R* and *p* values of the correlation analysis.

**FIGURE 6:**
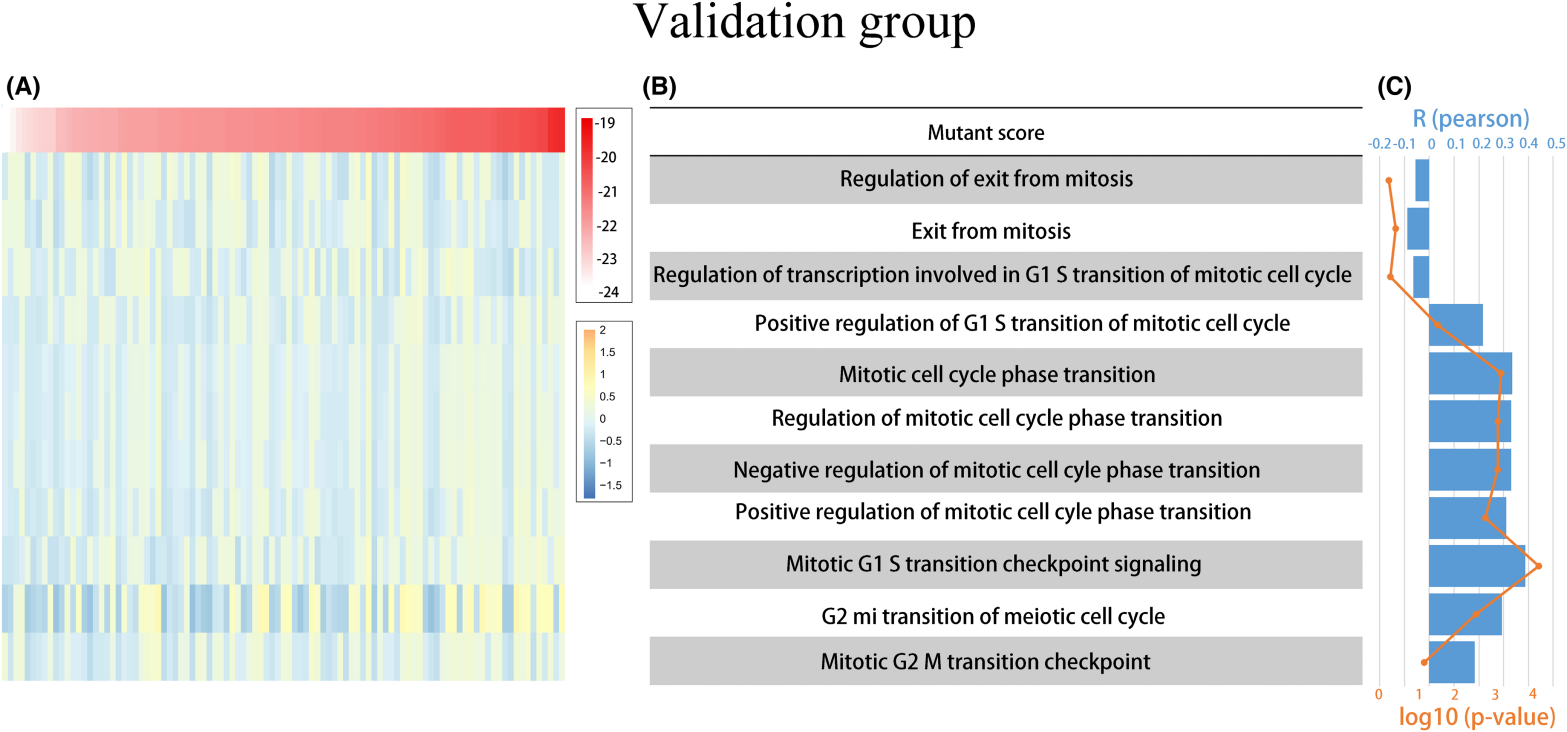
Correlation analysis of mutant scores with mitotic cell cycle enrichment scores. (A) A heatmap showing the expression of mutant fraction and mitotic cell cycle enrichment scores for each patient in validation groups, sorted from highest to lowest mutant fraction. (B, C) Right‐hand bar and line graphs showing the *R* and *p* values of the correlation analysis.

**FIGURE 7:**
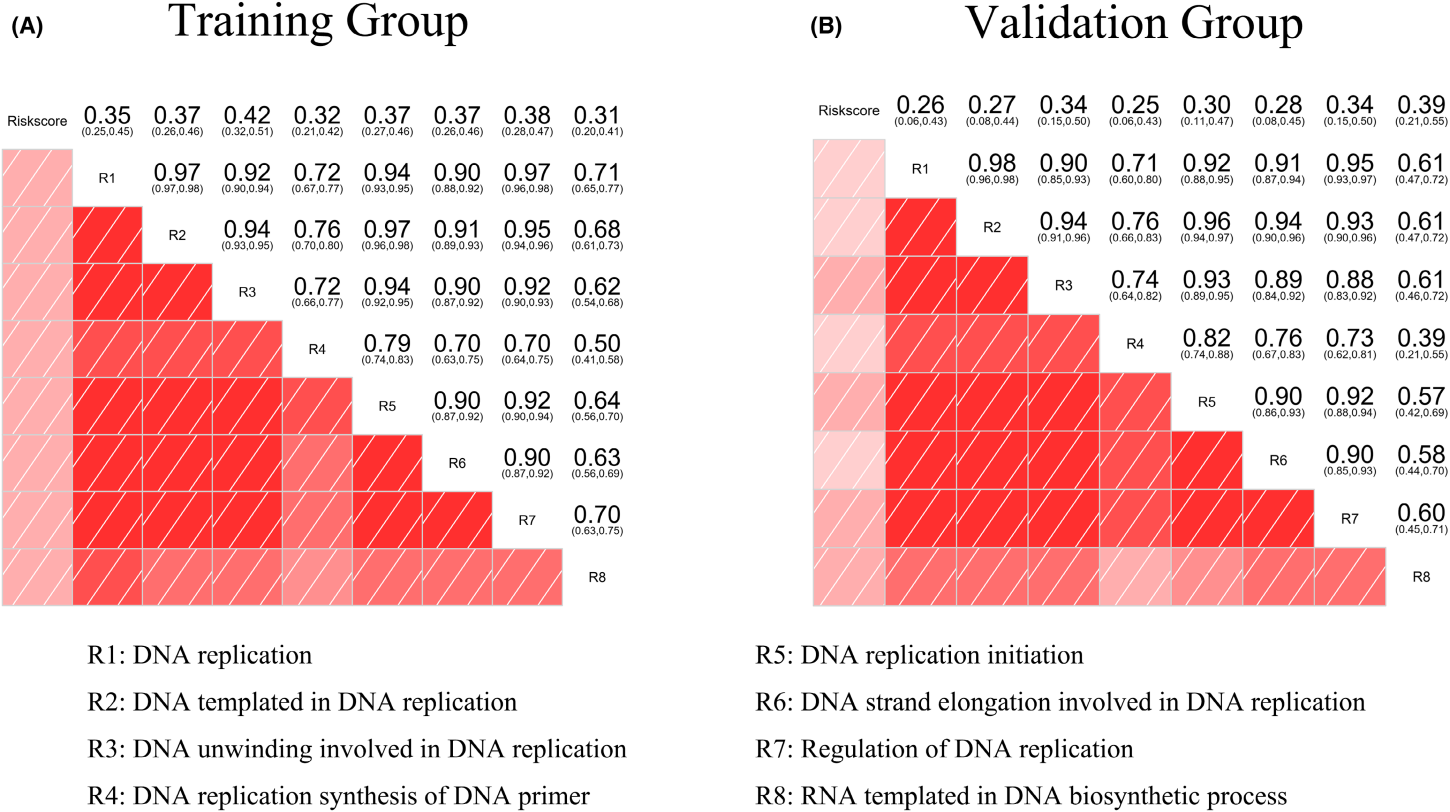
The correlation between mutant scores and DNA replication activities in the Training Group and Validation Group. (A, B) Correlation matrix of mutant scores and DNA replication‐related. The Upper right showed the correlation coefficient. The red parts represented a positive correlation. The correlation was tested by Pearson correlation analysis.

To further investigate the association between mutant scores and the phase transition of the mitotic cell cycle, we conducted GSVA on both the training and validation groups to calculate the enrichment scores of the mitotic cell cycle phase transition and its related regulatory processes. By analyzing the association between this enrichment and mutant scores, we observed a positive correlation between the expression of mutant scores and the majority of mitotic cell cycle‐related functions. These findings were validated in the training group, except for mitotic exit function and transcriptional regulation of the G1/S transition of the mitotic cell cycle. This comprehensive analysis suggests a strong association between the mutant fraction and the phase transition of the mitotic cell cycle, as well as its associated regulatory processes (Figures 5 and 6). Moreover, the progression of malignant tumor cells is intricately linked to DNA replication during the mitotic cell cycle. Therefore, we investigated the correlation between mutant scores and DNA replication processes. The results, as shown in Figure 7, exhibit strong correlations between mutation scores and fundamental aspects of DNA replication, such as DNA replication, its regulation, and the process of synthesizing RNA templates involved in DNA replication.

### 3.7 Strong predictive accuracy of the prediction models

**FIGURE 8:**
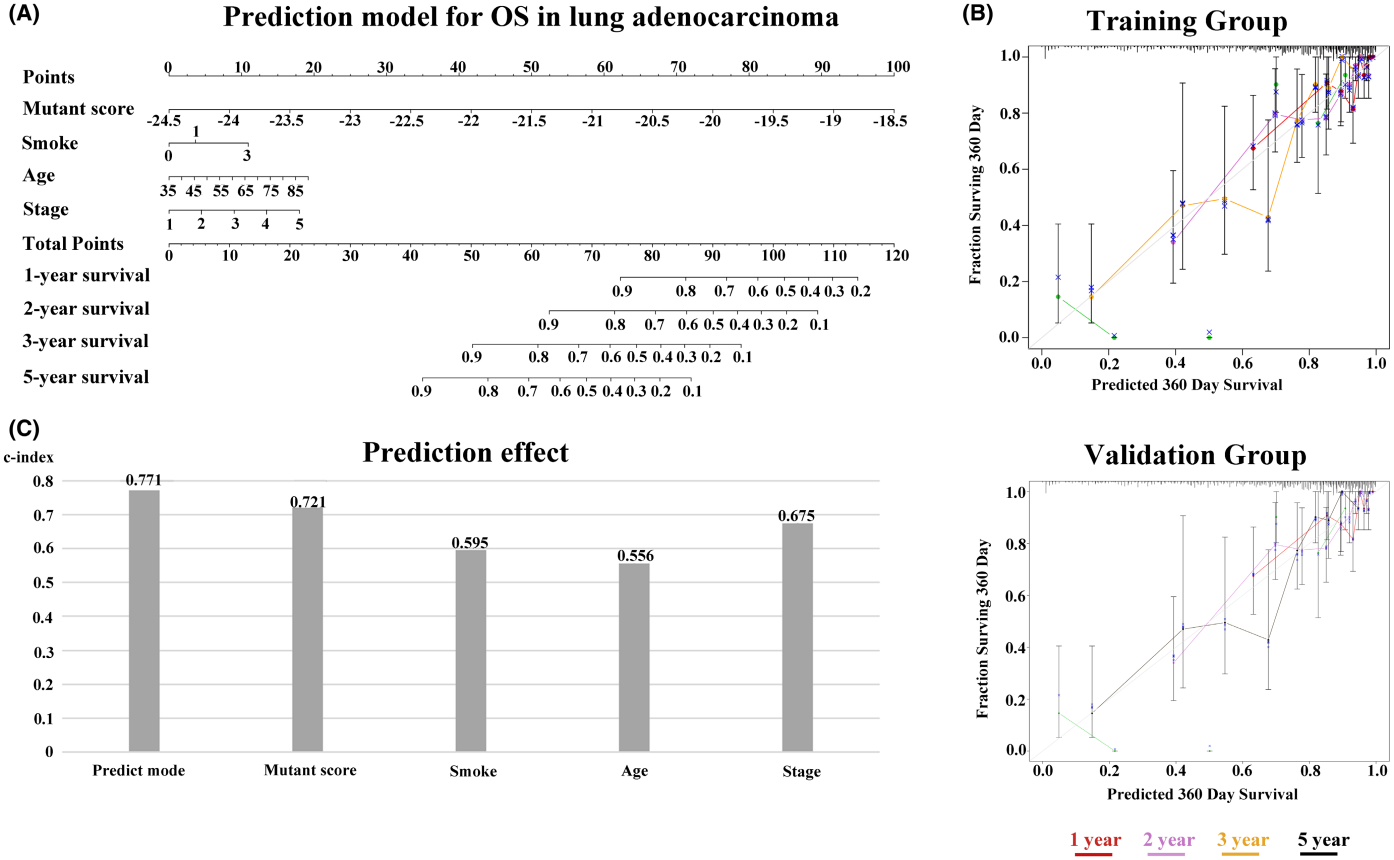
An individualized prediction model for patients with LUAD. (A) Nomogram accurately predicting 1‐, 2‐, 3‐, and 5‐year survival rates of patients with LUAD. (B) Calibration plots comparing predicted OS with actual OS in the training and validation groups. (C) Evaluation of the predictive effect of the individualized prediction model, mutant score, and clinical prognostic factors of LUAD on OS using the *C*‐Index. LUAD, lung adenocarcinoma; OS, overall survival. [Correction added on November 23, 2023 after first online publication. In Figure 8A, Riskscore has been corrected to read Mutant score in this version.]

To facilitate the clinical application of the prognostic prediction model, we constructed a personalized prediction model for OS that incorporates four independent predictors: mutant score, smoking status, age, and staging status. The nomogram presented in Figure 8A shows the estimation of 1‐, 2‐, 3‐, and 5‐year tumor survival probabilities for patients with LUAD (Figure 8A). To assess the accuracy of the prediction model, we compared the actual observations with the predictions using the nomogram and calibration curves in both the training and validation groups. The overlapping of the observed outcomes with the predicted probabilities indicates a satisfactory level of prediction accuracy (Figure 8B). Furthermore, the nomogram model showed a higher *C*‐index of 0.771 compared to the prediction model based on individuals alone (Figure 8C).

## 4 DISCUSSION

The investigation of metabolic processes in LUAD is essential for cellular life. Investigating the metabolic processes involved in LUAD not only provides an additional direction for the treatment of patients with LUAD but also facilitates better clinical decision‐making and improves the prognosis of patients by predicting specific metabolic processes involved in LUAD.

In our study, we developed and validated a prognostic prediction model that specifically focused on the phospholipid dephosphorylation metabolic process in LUAD. To achieve this, we conducted a long‐term follow‐up using the GEO database, with the training group followed for up to 2077 days and the validation group for up to 1693 days. Additionally, we used transcriptomic data from the tumor tissues of these patients for other correlation analyses. After translating 21,284 genetic markers from the training group into 7751 biological processes, we identified 114 biological processes that demonstrated prognostic value in both the training and validation groups. Among these processes, we screened for the phospholipid dephosphorylation metabolic process that was found to have the most significant independent prognostic value in *TP53* mutant LUAD. Furthermore, our findings highlighted alterations in this metabolic function between *TP53* mutant and wild‐type LUAD, which have the potential to impact the prognostic outcome in both cases. Based on the independent prognostic value of the phospholipid dephosphorylation process, we conducted LASSO‐COX downscaling analyses on the involved genetic markers. We identified the most representative 13 genetic markers that contribute to the characteristic genes of the TP53 mutant in LUAD. With these 13 candidate genes, we constructed a novel nomogram based on the mutant score. This nomogram enables the prognostic prediction of patients with LUAD based on their mutant expression levels. The nomogram used a combination of mutant score, smoking status, tumor stage, and age to effectively identify patients with high‐risk LUAD. When compared to the clinical factor model alone, the nomogram combined with the mutant score demonstrated higher predictive accuracy. This highlights that our nomogram serves as a fast, user‐friendly, and efficient tool for personalized prediction and treatment guidance.

Cell division plays a crucial role in the malignant progression of cancer, with cellular activity during mitosis being a central event in the life cycle.^20^ Several mitosis‐related genes, such as *CENP‐U*,^21^ *NCAPG*,^22^ and *NDC80* ^23^ have been implicated in the malignant progression of LUAD. Therefore, mitosis is a critical factor for LUAD, and our study revealed a close association between the mutant signature which from phospholipid dephosphorylation metabolic process and the phase transition of the mitotic cell cycle. The mutant score showed an active correlation with the phase transition of G1/S and G2/M of the mitotic cell cycle. Consequently, the mutant score can serve as an accurate response to the transition of the mitotic cell cycle phases and may also be predictive of *KRAS* mutations in LUAD. This study suggests that targeted therapies focusing on the dephosphorylation function of phospholipids could potentially hinder the progression of LUAD, providing a novel direction for mitogenic targeted drugs. Previously, clinical trials for such drugs, including aurora kinase family inhibitors, polo‐like kinase 1, Mps1, Eg5, CENP‐5, and APC/cyclosome complexes, have faced mostly negative results.^24^ Furthermore, considering that altered DNA replication is a significant characteristic of various cancers such as lung, liver, and gastric cancers and that therapeutic regimens targeting DNA replication proteins like CDC6 and RPA1 have demonstrated a significant slowdown effect on the progression of LUAD. Therefore, we conducted further investigations into the association between mutant scores and DNA replication. Our findings indicated a positive correlation between mutant scores and DNA replication, with a higher correlation observed for positive regulation of DNA replication compared to negative regulation (Table S1).^25^, ^26^, ^27^, ^28^

In this study, we aimed to investigate the difference in the biological process of phospholipid dephosphorylation between lung adenocarcinomas with TP53 mutant and TP53 wild‐type, which has not been explored before. Through our meticulous investigation, we identified 13 gene markers associated with this process. Further analysis allowed us to construct a mutation score. GO, GSEA, and GSVA analyses demonstrated a close correlation between the mutation scores derived from phospholipid dephosphorylation and transformative activity during the mitotic cell cycle phase. Thus, the present study may open new avenues for investigating the relationship between phospholipid dephosphorylation and LUAD, meanwhile, Our nomogram serves as a clinically applicable tool that focuses on the clinical parameters of LUAD. It intentionally excludes factors that would necessitate specialized analysis, such as tumor growth site, tumor volume, and metastatic status. While the tool may not be flawless, the clinical parameters involved are readily accessible, and the predictive probabilities align reasonably well with the actual outcomes, allowing the prediction model to be widely used.

The study has some certain limitations that should be acknowledged. Firstly, the size of sample which used in the study may be insufficient, which could potentially impact the accuracy of the model. To ensure the widespread applicability of the model, it is crucial to improve and expand both the accuracy index of the model and the clinical factors incorporated into it. Furthermore, due to the limited sample size, there were inconsistencies in the clinicopathological characteristics observed between the training and validation groups. The cost of sequencing the genes involved in calculating mutant scores adds to the financial burden on patients. Therefore, it is crucial that we persist in exploring better ways to enhance convenience and cost‐effectiveness for patients, thereby alleviating their financial strain.

In summary, this current study offers a tool for objectively assessing the probability of survival in patients with LUAD. Additionally, it provides a valuable reference for evaluating the activity of mitotic cell cycle phase transition in patients with LUAD.

## AUTHOR CONTRIBUTIONS

**Xu He:** Data curation (equal); resources (equal); validation (equal); visualization (equal); writing – original draft (equal). **HuaFu Zhou:** Funding acquisition (equal); methodology (equal); project administration (equal); writing – review and editing (equal). **qianyu huang:** Data curation (equal); visualization (equal). **Yue Li:** Data curation (equal); resources (equal).

## CONFLICT OF INTEREST STATEMENT

None of the authors have any conflict of interest to disclose.

## Supporting information


Table S1.

## Data Availability

All the data used or analyzed in this study are accessible from the corresponding authors upon request, and no additional ethics committee approval was required for their usage.
