# Supplementary material for: The mitotic cell cycle‐associated nomogram predicts overall survival in lung adenocarcinoma
Source: Cancer Med. 2023 Nov 6;12(23):21519–30. doi: 10.1002/cam4.6676 (PMC10726878; doi:10.1002/cam4.6676)
Supplement: Supplementary file 1 — Table S1. [file CAM4-12-21519-s001.docx]

| Mutant Score | Cor | P-value |
| --- | --- | --- |
| DNA Replication | 0.27244 | 1.74E-06 |
| Positive regulation of DNA replication | 0.306881 | 6.11E-08 |
| Negative Regulation of DNA replication | 0.279374 | 9.17E-07 |
|  |  |  |

Correlation of Mutant score with DNA replication
